# High prevalence of simian foamy virus infection of South American Indians

**DOI:** 10.1371/journal.ppat.1013169

**Published:** 2025-06-09

**Authors:** William M. Switzer, HaoQiang Zheng, Cláudia P. Muniz, Yi Pan, Hongwei Jia, Shaohua Tang, Anupama Shankar, Roxana Cintron, Jan Drobeniuc, Marcelo A. Soares, Walid Heneine

**Affiliations:** 1 Laboratory Branch, Division of HIV/AIDS Prevention, National Center for HIV/AIDS, Viral Hepatitis, STD, and TB Prevention, Centers for Disease Control and Prevention, Atlanta, Georgia, United States of America; 2 Departamento de Genética, Universidade Federal do Rio de Janeiro, Rio de Janeiro, Brazil; 3 Division of Viral Hepatitis, National Center for HIV/AIDS, Viral Hepatitis, STD, and TB Prevention, Centers for Disease Control and Prevention, Atlanta, Georgia, United States of America; 4 Programa de Oncovirologia, Instituto Nacional de Câncer, Rio de Janeiro, Brazil; Vaccine Research Center, UNITED STATES OF AMERICA

## Abstract

Simian foamy viruses (SFV) are retroviruses that widely infect nonhuman primates of New and Old-World origin and exhibit long-standing co-evolution with their hosts. Humans can acquire SFV from zoonotic exposures but are not known to be endemically infected and typically exhibit dead-end infections. South American Indian populations (Amerindians) historically have frequent contact with New World monkeys (NWM) and are endemically infected with the retrovirus human T-cell lymphotropic virus type 2 (HTLV-2) originally acquired before populating the Americas. We tested archived serum samples collected from numerous South American Indian persons (n = 1,998) in 1966–1997 from 18 tribes by validated ELISA and Western blot assays containing NWM SFV antigens. We also screened samples for HTLV-2 to compare SFV and HTLV-2 infection. We combined demographic and familial relationship data with the serologic results to evaluate transmission dynamics. We found a high SFV seroprevalence of 9.5% in 15 tribes compared to an HTLV-2 prevalence of 6.1% in 11 tribes. Testing of seropositive samples with Old World SFV antigen showed no reactivity confirming a NWM SFV origin. We found the odds of SFV and HTLV positivity increased with age and documented familial clustering of SFV among spouses and children. Serotyping showed co-circulation of SFV from different NWM species. Our results show that NWM SFV established longstanding prevalence among Amerindians post population of America. The high SFV prevalence will enable studies of disease association and human transmissibility to better understand the public health significance of SFV infections in humans.

## Author summary

Retrovirus emergence raises public health concerns as all human retroviruses, including HIV, originated from cross-species infections from nonhuman primates. Simian foamy viruses (SFV) are retroviruses that widely infect nonhuman primates of New and Old-World origin. Humans can acquire SFV from zoonotic exposures such as hunting, keeping nonhuman pets, and husbandry in zoos and research centers, but are not known to be endemically infected. We screened a large number of samples from eighteen South American Indian tribes (Amerindians) and identified a high SFV seroprevalence of 9.5%. We documented familial clustering of SFV among spouses and children and found serologic evidence of co-circulation of SFV from different New World primate species. The SFV prevalence was higher than that of the human T-cell lymphotropic virus type 2 retrovirus that is known to be endemic in Amerindians, and which has an ancient nonhuman primate origin in Africa. Our results demonstrate a high prevalence of SFV among Amerindians post population of America which will enable studies of disease association and human transmissibility to better understand the public health significance of SFV in humans.

## Introduction

Emerging viral zoonoses continue to receive heightened public health attention following the pandemic spread of SARS-CoV-2, and the Mpox and Ebola epidemics [1,2]. Viral zoonosis has also been recognized as the origin of human retroviruses [3–8]. Hunting and butchering of wild nonhuman primates (NHPs) infected with simian immunodeficiency virus (SIV) is thought to have sparked the human immunodeficiency virus (HIV) pandemic [4,7]. Similarly, the human T-lymphotropic viruses (HTLVs) types 1 and 2 originated independently from their simian analogs in Africa and spread globally [3,9]. HTLV-1 spread to the Caribbean and South America was facilitated by the slave trade [3]. HTLV-2 spread through infected humans out of Africa and Asia, across the Bering Strait over 15,000 years ago, and is currently endemic in most American Indian populations (Amerindians) in North, Central and South America [9]. The more recent discovery of two unique HTLVs, HTLV-3 and HTLV-4, among African bushmeat hunters originating from similar simian viruses suggests that such cross-species transmissions are not rare historical events [5,10–13].

NHPs are natural hosts to another retrovirus, the simian foamy viruses (SFVs). Unlike other simian retroviruses that only infect Old World NHPs, SFV widely infects both Old World and New World NHPs [14–17]. SFV demonstrates ancient co-evolution with its host with high genetic and antigenic divergence exhibited between disparate SFV variants from Old and New World NHPs [18,19]. Despite long-standing co-evolution in primates, humans are not known to be naturally infected with a human-specific foamy virus but are susceptible to zoonotic SFV infection [6,20]. Surveys have demonstrated transmission of SFV to persons who are directly exposed to NHPs, including Africans who hunt and butcher NHPs or workers at zoos and primate centers [6,20]. In these populations SFV seroprevalence ranged between 1–5%, while a higher prevalence is seen in persons who reported NHP bite injuries [6,20–23]. Phylogenetic analysis identified multiple Old World NHP species as the source of SFV infections in humans including chimpanzees, gorillas, baboons, mandrills, African green monkey, and macaques which collectively are referred to as Old World monkeys and apes (OWMAs) [6,20–23]. SFV infection of humans is characterized by persistent seropositivity and in many cases amplification or isolation of SFV from peripheral blood lymphocytes [6,20–23]. Although SFV is not known to be pathogenic in naturally infected NHPs, changes in the pathogenicity of simian retroviruses following cross-species infection are well documented as HIV emerged from benign SIV infections in the natural primate hosts [4]. To date, SFV-infected persons have not shown evidence of disease, although this finding is limited by the fact that these individuals have been identified in surveys of generally healthy populations [6,20–23]. Incidence of disease in SFV-infected persons may be low, may follow long latency periods, or may only result from specific SFV variants [6]. A case control study of 24 apparently healthy Cameroonian hunters infected with gorilla-type SFV showed evidence of anemia and hematological abnormalities that are of unclear significance [24]. Studies of human-to-human spread of SFV to date have not demonstrated familial clustering or transmission to sexual partners highlighting the difficulty of spread of SFV among humans [6,20–23]. Collectively, these data have suggested that SFV may represent benign dead-end infections in humans [6,20–23].

New World monkeys (NWMs) comprise at least 170 species divided into three families (*Cebidae, Atelidae*, and *Pitheciidae*) and 21 genera [18]. The phylogeny of SFV among NWMs generally mirrors its host suggesting ancient co-evolution, although some exceptions have been noted [14–16,18]. The genetic and antigenic divergence between Old World and New World SFV is significant with only 35% identity in the diagnostic group specific antigen (Gag) protein resulting in little or no serologic cross reactivity [14,15]. To ensure high detection sensitivity across SFV groups enzyme immunoassays (EIAs) and confirmatory western blot (WB) tests incorporate SFV antigens from either Old or New World NHPs [6,14,15]. Seropositivity in either test among humans can indicate infection with Old or New World SFV [6,25,26].

The Amazon forest overlaps eight countries in South America, including 58.1% in Brazil and 6.1% in Venezuela, and has the largest NHP diversity in the world with at least 120 species [27]. Historically, South American Indian populations residing in the Amazon forests have frequent contact with different monkeys from all three NWM families, including marmosets (*Callitrichinae*), spider monkeys (*Atelinae*), capuchins (*Cebinae*) and tamarins (*Callitrichinae*), either through hunting and butchering, or keeping them as pets, increasing their exposures to zoonoses of simian origin [27]. In addition, NWMs are common members of zoological collections and are frequently used in research [28]. A study of primate workers demonstrated WB seropositivity to NWM SFV providing the first evidence of susceptibility of humans to these infections [25,26]. Many, but not all, seropositive workers reported parenteral exposures from NWMs such as bites, needle stick injuries or scratches while others reported exposures to body fluids suggesting that parenteral contact alone may not be necessary for zoonotic transmission [25,26]. To better understand NWM SFV infection in natural settings, we conducted a large serosurvey of almost 2,000 Amerindian persons from 17 tribes in Brazil and one tribe in Venezuela. We also screened samples for HTLV-2 infection that is known to be endemic in this population [9,29]. We compared demographic and familial clustering for both viruses to gain insights into transmission dynamics and endemicity of both infections.

## Methods

### Ethics statement

This study was reviewed by the Centers for Disease Control (CDC), deemed research not involving human subjects, and was conducted consistent with applicable federal law and CDC policy (Protocol #6653) using anonymized participant specimens and information.

### Study population

**Fig 1:**
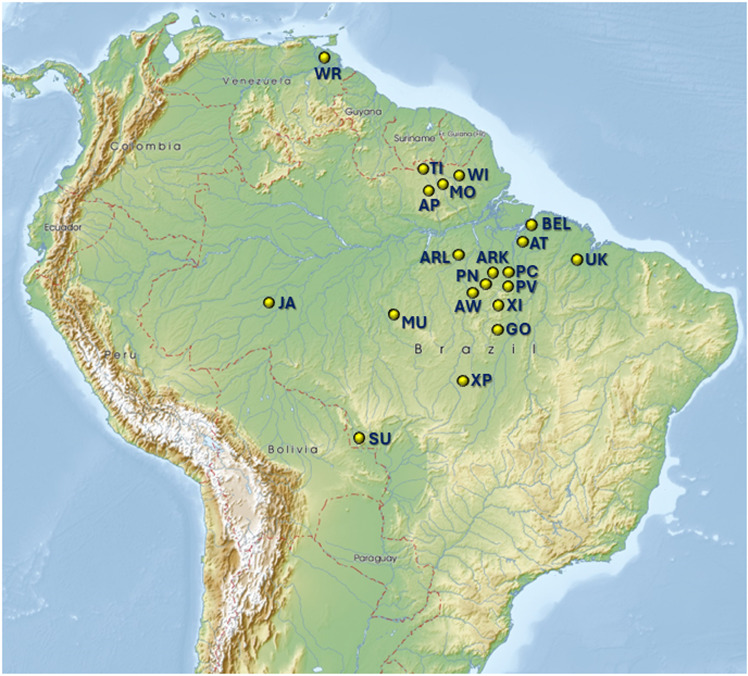
Topographical map showing approximate locations of South American indigenous populations in a study of simian foamy virus (SFV) and human T-cell lymphotropic virus (HTLV) prevalence (yellow dots). *Brazil:* Apalai (AP), Arara do Kurambê (ARK), Arara do Larangal (ARL), Arawéte (AW), Asuruni do Trocara (AT), Belém (BEL) Casa do Índio, Gorotire (GO), Jamamadi (JA), Molokopote (MO), Mundurucu (MU), Parakanã Novo (PN), Parakanã Velho (PV), Parakanã C (PC), Surui de Rondônia (SU), Tiriyó (T), Urubu-Kaapor (UK), Waiãpi (WI), Xikrin (XI), and Xingu Indigenous Park (XP); *Venezuela:* Warao (WR). Geographic map provided by mapswire.com under the Creative Commons BY 4.0 license (Free Maps of South America | Mapswire).

We conducted a retrospective study with sera collected from 17 Amerindian tribes from Brazil and one from Venezuela between 1966 – 1997 by Dr. Francis Black (Table 1 and Fig 1) [30–35]. The tribes in our study resided in the Amazon forests and included the Apalai (AP), Arara do Kurambê (ARK), Arara do Larangal (ARL), Arawete (AW), Asuruni do Trocara (AT), Gorotire (GO), Jamamadi (JA), Molokopote (MO), Mundurucu (MU), Parakanã Novo (PN), Parakanã Velho (PV), Parakanã C (PC), Surui de Rondônia (SU), Tiriyó (T), Urubu-Kaapor (UK), Waiãpi (WI), Warao (WR), and Xikrin (XI) [30–35]. Sera were also available from Amerindians residing at two health clinics and a national park in Brazil. Casa do Índio are health clinics for indigenous people created in 1968 by the former National Indian Foundation (Funai). Twenty-six sera were collected in 1987 from persons at a Casa do Índio in Belém (BEL), the capital of the State of Pará, Brazil. Ethnicity and demographic information were not available for these 26 persons. Serum samples were also collected from various Amerindian persons residing at another Casa do Índio (CD) between 1980 – 1983 but for which the location was not provided, including AP (n = 1), AT (n = 1), Galabi (GAL, n = 1), Gaviao (GAV, n = 4), G (n = 5), Karipuna de Rondônia (KAR, n = 9), Kayapo (KA, n = 5), MU (n = 9), Tembé (TE, n = 2), WI (n = 2), XI (n = 1), Yabeli (YA, n = 1). Sera collected in 1979 were also available from 19 persons at the Xingu Indigenous Park (XP) of which 9 were Caiabi, three were Suiá, two were Jurúna, and tribe names were not provided for five. Xingu Park is a national park on the upper Xingu River in the state of Mato Grosso, Brazil where indigenous people reside. Kinship was provided in records kept by Dr. Black. All sera were stored at -20°C until tested. Serum samples were linked to demographic data by using anonymous numerical codes.

**Table 1:** South American indigenous study population and prevalence of simian foamy virus (SFV) and human T-lymphotropic virus (HTLV)^1^.

Tribe	Tribe ID	Country	No. Persons (Est. % of tribal population)	Sample Year(s)	Median age, range	Sex (% M,F,U)^2^	SFV Prevalence (No. positive, %, 95% CI)	HTLV Prevalence^3^ (No. positive, %, 95% CI)
Apalai	AP	Brazil	80 (26.5)	1977, 1983, 1988	23.0, 6-67	38.7, 31.3, 30.0	0, 0 (0.1, 5.7)	0, 0^5^ (0.1, 5.7)
Arara do Kurambê	ARK		18 (85.7)	1985, 1986	11.0, 2-46	50.0, 50.0, 0.0	**2, 11.1** (1.9, 36.1)	0, 0 (0.5, 21.9)
Arara do Larangal	ARL		47 (90.4)	1985	17.0, 2-71	40.6, 39.6, 19.8	**1, 2.1** (0.1, 12.7)	**1, 2.1** (0.1, 12.7)
Araweté	AW		101 (57.7)	1986	24.0, 1-70	37.6, 33.7, 28.7	**5, 5.0** (1.9, 11.8)	0, 0 (0.1, 4.6)
Asuruni do Trocara	AT		109 (77.9)	1984	17.5, 2-80	51.4, 47.7, 0.9	**7, 6.4** (2.8, 13.2)	0, 0 (0.1, 4.2)
Gorotire	GO		17 (4.3)	1979, 1981, 1982	26.5, 2-47	23.5, 64.7, 11.8	**2, 11.8** (2.1, 37.8)	**6, 35.3** (15.3, 61.4)
Jamamadi	JA		42 (63.6)	1986	21.0, 3-65	38.1, 47.6, 14.3	**5, 11.9** (6.0, 29.2)	0, 0 (0.2, 10.4)
Molokopote	MO		15 (65.2)	1978	7.5, 1-25	40.0, 13.3, 46.7	0, 0 (0.6, 25.4)	0, 0 (0, 25.4)
Mundurucu	MU		197 (5.9)	1985	15.0, 1-75	47.7, 42.6, 9.1	**3, 1.5** (0.4, 4.7)	**1, 0.5** (0, 3.2)
Parakanã C	PC		125 (90.6)	1984	17.0, 1-60	52.8, 47.2, 0.0	**25, 20.0** (14.4, 29.4)	**9, 7.2** (3.5, 13.5)
Parakanã Novo	PN		33 (100)	1977, 1978	12.0, 2-45	57.6, 36.7, 6.1	**2, 6.1** (1.1, 22.7)	**1, 3.1** (0.2, 17.5)
Parakanã Velho	PV		99 (81.8)	1977, 1978, 1981	28.5, 1-67	11.1, 16.2, 72.7	**7, 7.1** (3.2, 14.6)	**4, 4.0** (1.3, 10.6)
Surui de Rondônia	SU		42 (14.4)	1987	20.0, 3-50	47.6, 52.4, 0.0	0, 0 (0.2, 10.4)	0, 0 (0.2, 10.4)
Tiriyó	TI		224 (31.9)	1966, 1977, 1978, 1980	24.0, 2-71	17.8, 15.2, 67.0	**14, 6.3** (3.6, 10.6)	**6, 2.7**^**6**^ (1.1, 6.0)
Urubú-Kaapor	UK		146 (28.2)	1984	19.0, 2-70	55.5, 43.8, 0.7	**10, 6.9** (3.6, 12.6)	**1, 0.7**^**7**^ (0, 4.4)
Waiãpi	WI		179 (47.7)	1980	17.0, 2-45	17.8,17.3,64.8	**38, 21.2** (15.6, 28.1)	**1, 0.6**^**8**^ (0, 3.6)
Xikrin	XI		280 (54.2)	1970, 1972, 1976, 1978, 1979, 1981, 1986, 1994	18.0, 1-84	48.2, 49.3, 2.5	**46, 16.4** (12.4, 21.4)	**86, 30.7** (25.4, 36.5)
Warao	WR	Venezuela	151 (6.3)	1986	22.0, 5-72	17.9, 17.9, 64.2	**13, 8.6** (4.8, 14.6)	**1, 0.7** (0, 4.3)
***Subtotal***	18		**1,905 (51.8)**	**1966-1994**	**18.0, 1-84**	**37.4, 35.2, 27.5**	**180, 9.5 (8.3, 10.9)**	**117, 6.1 (5.1, 7.3)**
**Location**	**Location ID**	**Country**	**No. Persons** **(Est. % of tribal population)**	**Sample Year(s)**	**Median age, range**	**Sex (% M,F,U)**	**SFV Prevalence**	**HTLV Prevalence**
Casa do Índio Belém^8^	BEL	Brazil	26 (N/A)	1997	55.0, 16-50	15.4, 11.5, 73.1	**4, 15.4** (5.1, 35.7)	**2, 7.7** (1.4, 26.6)
Casa do Índio^9^	CD		40 (N/A)	1980, 1981, 1982, 1983	N/A	N/A	**4, 10.0** (3.3, 24.6)	**3, 7.5** (2.0, 21.5)
Xingu Indigenous Park^10^	XP		19 (2.3)	1979	N/A	N/A	0, 0 (0.5, 20.9)	0, 0 (0.5, 20.9)
***Subtotal*** ^***11***^	3		**85 (N/A)**	**1979-1997**	**55.0, 16-50**	**15.4, 11.5, 73.1**	**8, 9.4 (4.4, 18.2)**	**5, 5.9 (2.2, 13.8)**
***Total***	21	Brazil and Venezuela	**1990 (49.2)**	**1966-1997**	**18.0, 1-84**	**36.0, 33.8, 30.2**	**188, 9.5 (5.9, 14.8)**	**122, 6.1 (2.8, 12.3)**

1. Seroprevalence determined by Amerindian tribe or location when tribe was not reported, or multiple tribes were present at the location. Seroprevalence for persons with longitudinal specimens reflect testing western blot (WB) positive for HTLV or SFV at any timepoint.

2. M, male; F, female; U, unknown; N/A, not available.

3. There were not sufficient specimen volumes for 84 specimens from 51 persons for HTLV EIA testing. Five samples from four persons were HTLV EIA reactive but did not have sufficient specimen volumes for HTLV WB testing.

4. Three samples were HTLV EIA reactive but did not have sufficient specimen volumes for HTLV WB testing.

5. Four samples were HTLV EIA reactive but did not have sufficient specimen volumes for HTLV WB testing.

6. Two samples were HTLV EIA reactive but did not have sufficient specimen volumes for HTLV WB testing.

7. Six samples were HTLV EIA reactive but did not have sufficient specimen volumes for HTLV WB testing.

8. Belém is an urban city on the Amazon River in the Pará state of Brazil. Tribe names were not provided.

9. Indian clinic visited by indigenous people from multiple tribes, including Gavião (n = 4), GO (n = 5), MU (n = 9), Tembé (n = 2), WI (n = 1), AP (n = 1), AT (n = 1), Galibi (n = 1), Karipuna de Rondônia (n = 9), Kayapo (n = 5), Xikrin (n = 1), Yabeli (n = 1). Location not provided.

10. Nine were Caiabi, three were Suiá, two were Jurúna; tribe names were not provided for five members at Xingu Park.

11. Demographic totals based on only persons at Casa do Índio, Belém.

### Serology

SFV serology was done using two sequential EIAs followed by confirmation of EIA reactivity with western blot (WB) testing. We first screened serum samples for antibodies to NWM SFV using a validated EIA for antibodies to NWM SFV as previously described [14–16,25]. This EIA contains whole-virus tissue culture antigens from two divergent NWM SFV isolates from a marmoset (*Callithrix jacchus*, SFVcja) and a spider monkey (*Ateles* species, SFVasp) in a single well to broadly detect NWM SFV antibodies. The SFV isolates were expanded in canine thymocytes (Cf2Th) so an additional well including uninfected Cf2Th cell lysates was used to control for non-specific reactivity [14–16,25]. EIA-reactive samples were retested and if still reactive were then tested in a new EIA using recombinant SFVcja and SFVasp Gag proteins (recGag) prepared by Biomatik (https://www.biomatik.com/). Preparation of the recGag proteins included optimization of the nucleotide sequences for expression in *E. coli* with a histidine (His) tag for purification with an anti-His antibody. Purity of the recGag proteins was estimated at > 85% using Coomassie blue-stained SDS-PAGE. This new EIA was validated using sera and plasma specimens from both human and NHPs, including US blood donors (n = 102), HIV-infected persons (n = 86), HTLV-infected persons (n = 47), humans and simians infected with Old World NHP SFV (n = 32), SFV-infected prosimians (n = 2), and SFV WB-negative (n = 27) and positive NWM (n = 57) from each of the three Platyrrhini families (S1 Table). Serum samples were diluted 1:100 and tested in the same format as the first EIA screening test [14,16,25]. Samples that were repeatedly reactive in the second EIA were tested using a validated WB assay containing both SFVcja and SFVasp antigens in cultured Cf2Th cells as previously described [14,15,25]. Seroreactivity to both Gag p68 and p72 precursor proteins with an absence of similar reactivity to antigen from SFV-uninfected Cf2Th cells was interpreted as WB seropositive and evidence of SFV infection. Specimens showing reactivity to only one Gag protein or no reactivity to either Gag protein were considered negative. Persons with longitudinal samples were considered SFV seropositive if any of their samples were WB positive. We also tested NWM WB-positive samples with sufficient volumes of SFV EIAs and WB tests containing Old World SFV antigens as previously described to exclude cross-reactivity with Old World SFV [6]. Curated negative and SFV positive controls were tested in each EIA and WB test to ensure the quality of each assay run.

To gain insights into the NWM species origin of the infection among the SFV-seropositive individuals, we developed and used a new SFV serotyping EIA to determine if seropositivity was more like that of SFV from marmosets (*Callithrix jacchus*) or spider monkeys (*Ateles* species). Samples were tested at a 1:100 dilution in two independent EIA assays containing single recGag proteins from either marmoset (SFVcja) or spider monkey (SFVasp) following the EIA protocol for the combined recGag antigens described above. Reactivity was determined using a 0.1 OD cutoff. Samples showing a higher reactivity to SFVcja were considered marmoset-like SFV infection while those showing higher reactivity to SFVasp were considered spider monkey-like SFV infection. Samples with equal reactivity to both antigens were considered untypeable. The assays were first evaluated using plasma from 28 SFV-negative NWMs (owl monkey (n = 9), spider monkey (n = 3), marmoset (n = 9), capuchin (n = 2), tamarin (n = 2), and squirrel (n = 3)) and 108 SFV-positive NWM from three subfamilies: 1. *Atelidae* (spider monkeys (*Ateles* species (n = 25)), howler monkeys (*Alouatta* species (n = 9)), wooly monkeys (*Lagothrix* species (n = 6)); 2. *Cebidae* (marmosets (*Callithrix* species (n = 10)), owl monkeys (*Aotus* species (n = 10)), capuchins (*Cebus* species (n = 25)), tamarins (*Leontopithecus* species (n = 6)), *Saguinus* species (n = 2)), and squirrel monkeys (*Saimiri* species (n = 22)); 3. *Pitheciidae* (uakari monkeys (*Cacajao melanocephalus* (n = 6)) and *Cacajao rubicundus* (n = 3)),saki monkeys (*Chiropotes* species (n = 1)) and (*Pithecia pithecia* (n = 9)) (S2 Table).

We also tested the sera for HTLV using commercial EIA and WB kits as previously described to compare HTLV and SFV infections in the same Amerindian population [36]. Briefly, a commercial EIA that contains HTLV-1 and -2 antigens (HTLV-I/II ELISA 4.0; MP Biomedicals, Santa Ana, CA) was used. Samples with OD values above the manufacturer’s pre-determined assay cut-off were re-tested in duplicate when sample volume was sufficient. Repeat reactive samples were further tested by HTLV-1/2 WB testing (HTLV Blot 2.4; MP Biomedicals, Santa Ana, CA) and interpreted according to the manufacturer’s instructions. Specimens that were reactive to both Gag p24 and envelope GD21 proteins were considered WB-positive. HTLV typing was determined by reactivity to rgp46-I (MTA-1) for HTLV-1 and rgp46-II (K55) for HTLV-2. Samples that were WB-positive but not reactive to either the MTA-1 or K55 peptides were considered HTLV-positive, but untypeable. Samples not meeting these criteria were considered HTLV negative. Persons with longitudinal samples were considered HTLV seropositive if any of their samples were WB positive. This HTLV testing strategy has been successfully utilized in previous studies to identify different HTLV infections, including the highly divergent HTLV-3 and -4 [12,13,36].

### Statistical and clustering analysis

Receiver operator curve (ROC) analysis of SFV EIA sensitivity, specificity, accuracy, and inferred assay cutoffs were determined using MedCalc Statistical Software version 22.006 (MedCalc Software Ltd, Ostend, Belgium; https://www.medcalc.org; 2023). The Wilson score method was used to estimate the 95% confidence intervals of SFV and HTLV prevalence using a normal approximation for the binomial proportion.

We conducted univariate and multivariate analyses using logistic regression to compare the magnitude and significance of sex and age available for most participants (Table 1). We excluded individuals with missing sex information from the analysis to ensure the clarity and interpretability of sex-related analyses. While the proportion of missing sex was similar across groups, their inclusion could introduce uncertainty in sex-specific comparisons, particularly in evaluating potential associations with SFV or HTLV seropositivity. Any variable with a *p* value <0.25 in the univariate model was included in the multivariate model. A *p* value <0.05 was considered statistically significant. We evaluated the significance of SFV and HTLV-2 infection rates overall and among different tribes using McNemar’s test [37].

We also evaluated if age was associated with SFV or HTLV transmission between spouses by determining mean ages and the standard deviation of the mean age, and the minimum and maximum ages of the spouses. For this analysis, we combined spousal SFV and HTLV data from all tribes reporting spouse data. We evaluated four different spousal infection status combinations, including (1) both male and female partners were infected; (2) both male and female partners were not infected; (3) males were infected, but females were not infected; and (4) males were not infected, but females were infected. Persons ≤ 10 years old (yo) were considered children and were excluded from the spousal analysis. Linear regression models were applied to compare the ages for males and females and the difference between spouse ages in the four different scenarios. In addition, we used MicrobeTrace, a bioinformatics tool for analysis of pathogen transmission, to analyze clustering of SFV and HTLV seropositivity in tribes with available familial relationship (parent and child) data to further explore potential viral transmission [38].

We performed an agreement analysis using Cohen’s kappa statistic to evaluate agreement between the two SFV EIAs and improvement in assay accuracy using sequential EIA testing [39]. For the agreement analysis, we used the inferred assay cutoffs and WB results as the gold standard for evidence of SFV infection. We used for this analysis the simian and human samples that were utilized in the validation of the new recGag EIA as described above. A kappa value from 0.2 to 0.4 indicates fair agreement, 0.4-0.6 moderate agreement, 0.6 to 0.8 good agreement and greater than 0.8 excellent agreement [40].

We used SAS version 9.4 (SAS Institute, Cary, NC) to perform most statistical analyses. Fisher’s exact test was applied to evaluate an association of SFV/HTLV infection using MedCalc.

## Results

**Characteristics of study population.** Table 1 provides the demographic data available for each indigenous population and for one Indian clinic. Overall, ages ranged from 1–84 yo with a median of 18 yo. There were nearly equal proportions of males, females, and persons for whom sex was not available (33.85, 36.05, 30.2%, respectively). We also provide in Table 1 the estimated percentage of the total Amerindian population for each tribe in our study at the time of specimen collection, which ranged from 2.3% for Xingu Park to 100% for the Parakanã Novo.

**SFV recGag EIA serology and sequential EIA validation.** Fifty-six NWM SFV WB-positive samples and one sample from a Pithecia monkey were positive and negative in the NWM SFV recGag EIA, respectively (S1 Table). All NWM SFV WB-negative, OWMA SFV WB-positive, prosimian SFV WB-positive, U.S. blood donor, and HTLV WB-positive samples tested negative using the NWM SFV recGag EIA. Two HIV sera from African patients were EIA reactive to NWM SFV recGag antigens with ODs close to the assay cutoff of 0.231 (0.244 and 0.458). Both specimens showed high background reactivity in the SFV NWM WB assay and were seronegative in the OWMA SFV EIA and WB (not shown). Thus, the new SFV NWM recGag EIA had a sensitivity, specificity, and accuracy of 98.3% (95% CI 90.6-99.9%), 99.3% (95% CI 97.6-99.9%), and 99.2% (95% CI 97.5-99.8%), respectively.

We next evaluated the serial SFV serology testing design using Cohen’s kappa statistic. The kappa values for EIA assay agreement with WB results were highest with sequential specimen testing using the whole SFV lysate EIA followed by the SFV recGag EIA (0.96; 95% CI 0.9150, 1.000) compared to only using a single EIA (0.8897, 95% CI 0.8134, 0.9680 or 0.8121, 95% CI 0.7238, 0.9003, respectively). These kappa analysis results showing excellent agreement strongly support our usage of serial EIA testing followed by WB confirmation for SFV antibody detection.

**Fig 2:**
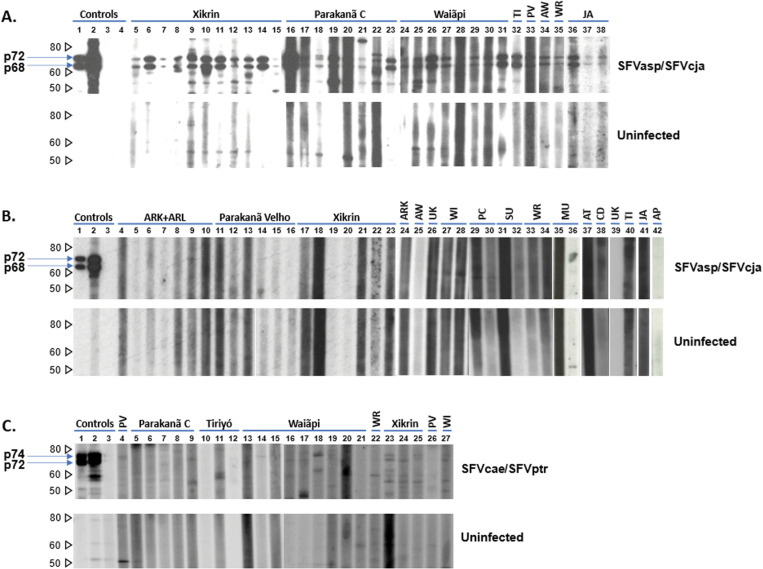
Detection of antibodies to New World monkey (NWM) simian foamy virus (SFV) among Amerindian persons. A. Western blot (WB) seroreactivity to SFV antigens from a spider monkey (*Ateles* species, SFVasp) and a common marmoset (*Callithrix jacchus*, SFVcja) in the upper panel and not to the uninfected canine thymocyte (Cf2Th) control antigens in the lower panel. Lanes 1 and 2, positive control sera from SFV-infected spider (*Ateles* species) and capuchin (*Cebus apella*) monkeys, respectively; lane 3, blank; lane 4, pedigreed negative US blood donor; lanes 5-15, Xikrin; lanes 16-23, Parakanã C; lanes 24-31, Waiãpi; lane 32, Tiriyó (TI); lane 33, Parakanã Velho (PV); lane 34, Arawéte (AW); lane 35, Warao (WR); lanes 36-38, Jamamadi (JA). B. Representative negative human NWM SFV WB results. Lanes 1 and 2, positive control sera from SFV-infected spider (Ateles species) and capuchin (Cebus apella) monkeys, respectively; lane 3, pedigreed negative US blood donor; lanes 4-10, Arara do Kurambê (ARK) and Arara do Larangal (ARL); lanes 11-16, Parakanã Velho; lanes 17-23, Xikrin; lane 24, ARK; lane 25, Arawéte (AW); lane 26, Urubu-Kaapor (UK); lanes 27-28, Waiãpi (WI); lanes 29-30, Parakanã C (PC); lanes 31-32, Surui de Rondônia (SU); lanes 33-34, Warao (WR); lanes 35-36, Mundurucu (MU); lane 37, Asuruni do Trocara (AT); lane 38, Casa do Índio (CD, location not reported); lane 39, Urubu-Kaapor (UK); lane 40, Tiriyó (TI; lane 41, Jamamadi (JA); lane 42, Apalai (AP). C. Absence of strong cross-reactivity to Old World monkey and ape (OWMA) SFV from African green monkey (AGM, *Chlorocebus aethiops*, SFVcae) and chimpanzee (*Pan troglodytes*, SFVptr) SFV-infected humans and not to uninfected Cf2Th control antigens in the lower panel. Lanes 1 and 2, positive control sera from SFV-infected AGM and chimpanzee, respectively; lane 3, pedigreed negative US blood donor; lane 4, Parakanã Velho (PV); lanes 5-9, Parakanã C; lanes 10-12, Tiriyó; lanes 13-21, Waiãpi; lane 22, Warao (WR); lanes 23-25, Xikrin; lane 26, Parakanã Velho (PV); lane 27, Waiãpi (WI). Lanes 23, 25, and 27 show weak OWMA SFV seroreactivity. Molecular markers in kDa are provided on the left and the location of the diagnostic 68/72 and 74/72 kDa NWM and OWMA SFV Gag doublet proteins, respectively, are shown with blue arrows. Results in the upper and lower panels in each figure are from the same film with the same exposure time.

**High seroprevalence of SFV.** A total of 2,752 sera from 1,990 persons were tested in the first EIA, of which 669 (24.3%) were reactive. Of the 669 samples, 366 were repeatedly reactive (54.8%) in the 2^nd^ SFV EIA and three did not have enough sample volume for repeat testing. WB confirmation of SFV seroreactivity was seen in 221/302 (73.2%) samples from 188 persons giving a total SFV prevalence of 9.5%. SFV antibodies were detected in 15/18 (83.3%) Amerindian tribes from Brazil and Venezuela with the prevalence ranging from 1.5- 21.2% (Table 1). SFV infection was found among Amerindian tribes that populate a large area about 2.5 million square kilometers in Brazil and extending further north to Venezuela among the Warao and in the urban city of Belém (Fig 1). We also detected 10.0 – 15.4% SFV seroprevalence in two Casa do Índio in Brazil. The clinic in Belém with the higher SFV prevalence did not provide the tribe names for persons living there, while in the second clinic one person each of the Yabeli (1/1, 100%), Karipuna de Rondônia (1/9, 11.1%, Waiãpi (1/1, 100%), Gavião (1/4, 25%), and Xikrin (1/1, 100%) tribes were SFV-seropositive. All persons from the Apalai, Molokopote, and Surui de Rondônia tribes were SFV-negative. Likewise, none of the three tribes in Xingu Park tested SFV-positive. Fig 2A provides examples of the range of NWM SFV seropositivity observed in specimens from Amerindian persons in our study. These results show NWM SFV infection is widespread in Brazil, including in one urban setting, and is also present in Venezuela. The earliest SFV were present in 1966 in the Tiriyó, while the most recent SFV were seen in 1994 in the Xikrin. The SFV prevalence in our study population was 8.2% for samples collected during 1966–1974, 7.4% for samples collected 1976–1985, and 7.5% for samples collected 1986–1997. These results demonstrate persistent NWM SFV infection across three decades for some Amerindian tribes. Fig 2B shows examples of specimens from tribes with seronegative results.

We next tested 186 NWM SFV seropositive serum samples available from 179 persons with our OWMA SFV tests to exclude cross reactivity to OWMA antigens. Of the 186 sera, 181 (97.3%) were negative and five were weakly reactive in the OWMA SFV testing demonstrating the predominance of NWM SFV infection. Fig 2C shows examples of the negative WB results and three of the weakly reactive specimens (lanes 23, 25, and 27). These findings show the observed SFV seroreactivity is specific to NWM SFV.

**Serotyping reveals human infection with different SFV variants.** Validation of the NWM SFV serotyping assay showed that 83.8% (31/37) and 73.1% (38/52) of *Atelidae* and *Cebidae* seropositive samples were correctly serotyped using the SFVasp and SFVcja-specific assays, respectively, highlighting the validity of this approach (S2 Table). One seropositive *Atelidae* sample (1/37, 2.7%) and nine seropositive *Cebidae* samples (9/51, 17.7%) were misclassified as marmoset-like (SFVcja) or spider-monkey-like (SFVasp) SFV, respectively. Five seropositive *Atelidae* samples (5/37, 13.5%) and five seropositive *Cebidae* samples (5/51, 9.8%) were untypeable. We also evaluated the specificity of the serotyping assays using 19 samples from the *Pitheciidae* subfamily that is phyletically distant from the *Atelidae* and *Cebidae* SFV, but closer genetically to the *Cebidae* SFV [14,18]. Of these 19 samples, 52.6% were SFVcja-like, 10.5% were SFVasp-like, and 36.8% were untypeable, highlighting the lower discrimination of these assays on divergent SFV infections in this subfamily. All 28 seronegative monkey samples were nonreactive.

Serotyping of 209 SFV seropositive samples from Amerindian persons showed that 55 (26.3%) were infections with spider monkey-like SFV, 10 (4.8%) were marmoset-like SFV and 122 (58.4%) were untypeable (S3 Table). Samples from 22 (10.5%) persons were nonreactive to the individual SFV recGag proteins, which may be related to the use of one half the antigen concentration in the serotyping tests. These results suggest that the SFV infections among the Amerindian persons are not due to a single SFV variant from a NWM species but more likely to multiple SFVs from different neotropical monkeys.

**SFV prevalence higher than HTLV-2.** To compare SFV and HTLV infections in this population we tested specimens with sufficient volume for HTLV infection. Of the 2,644 remaining specimens from 1,936 persons, 444 sera (16.8%) from 309 persons (16.0%) were HTLV EIA reactive. Of the 444 samples, adequate specimen volumes were available for 423 specimens from 286 persons for WB testing. HTLV testing found 223 sera (52.7%) from 122 persons were HTLV-positive giving a total prevalence of 6.1% which is significantly lower than the 9.5% SFV prevalence (*p* < 0.0001). Of the 223 WB-positive specimens from 122 persons, 190 samples from 98 persons (98/1936, 5.1%) were HTLV-2, one person each from the Parakanã C, Warao, and Xikrin tribes were weak HTLV-1 (3/1936, 0.2%), and 30 samples from 23 persons (23/1936, 1.2%) were HTLV-positive but untypeable. Most of the untypeable persons were from the Parakanã C tribe (n = 8, 53.3%). The HTLV prevalence in individual tribes ranged from 0.0 – 35.3%, confirming the endemicity of HTLV-2 in this study population is consistent with previous reports [9,41–45]. The HTLV prevalence in our study population was 16.2% for samples collected during 1966–1974, 6.4% for samples collected 1976–1985, and 8.4% for samples collected 1986–1997. Statistical comparison of SFV and HTLV infections rates by tribe demonstrated that five tribes had significant infection rate differences, including the Parakanã C (*p* = 0.0041), Urubu-Kaapor (*p* = 0.0067), Waiãpi (*p* < 0.0001), Warao (*p* = 0.0013), and Xikrin (*p* < 0.0001), with all except the Xikrin showing a higher prevalence of SFV than HTLV.

**Dual SFV and HTLV-2 infections.** We found 18 persons from five tribes who were dually infected with SFV and HTLV, giving an overall co-infection rate of 1.2%. These persons included 8 female and five male Xikrin aged 5–66 yo, two Parakanã Velho (sex and age not available), one Tiriyó (sex and age not available), one 35 yo Gorotire female, and one 7 yo Arara Larangal female.

**Association of SFV or HTLV infection with age but not sex.** For this analysis we combined HTLV-2, HTLV-1, and HTLV-untypeable into HTLV-positive. The mean and median ages for SFV seropositivity in males and females was 27.1 and 26.0 and 26.8 and 23.5 y, respectively. The age range for male and female SFV seropositivity was 2–60 and 1–75 yo, respectively. Twenty-two children ≤ 10 yo were SFV-positive, including 13 girls and 9 boys. The mean and median ages for HTLV seropositivity in males and females was 25.1 and 23.0 and 30.4 and 32.0 yo, respectively. The age range for male and female HTLV seropositivity was 2–84 and 3–66 yo, respectively. Sixteen children ≤ 10 yo were HTLV-positive, including 8 girls and 8 boys. More females were infected with SFV or HTLV than males (11% (74/673) and 8.4% (60/716) vs 9.8% (66/673) and 5.6% (40/716), respectively). Similarly, there were more persons for whom sex was not available with SFV vs HTLV infection (8.9% (54/601) vs 2.7% (16/601), respectively). Our results show similar age characteristics for both SFV and HTLV seropositivity with higher mean and median ages seen for HTLV-infected women.

Statistical modeling results for associations of SFV and HTLV infection with sex and age are presented in Table 2. For SFV infection, both the univariate and multivariate models showed that for every five years increment in age at sampling the odds of SFV positivity increases by 1.12 (95% CIs = 1.06 - 1.18 and 1.05 - 1.18, *p = *0.0001 and *p* = 0.0003, respectively). Likewise, for every 5 years increment in age at sampling increased the odds of HTLV infection in both models by about 1.15 and 1.18 (95% CIs = 1.1 - 1.2 and 1.12 – 1.25, p < 0.0001, respectively). Sex was not associated with SFV and HTLV infection in both models. Our findings indicate that age is associated with an increased odds for both SFV and HTLV infection in Amerindian persons likely resulting from longer exposures to the viruses.

**Table 2:** Association of sex and age with simian foamy virus (SFV) and human T-lymphotropic virus (HTLV) infection in South American Indian persons.

Univariate model						
	SFV			HTLV		
***Variable***	***Odds ratio***	***95% CI*** ^***1***^	***P value***	***Odds ratio***	***95% CI***	***P value***
**Sex**			0.158			0.222
* Female*	1.35	0.89, 2.03		1.85	0.69, 4.96	
* Male (Reference)*						
**Age at sampling (5 years increase)**	1.12	1.06, 1.18	**0.0001**	1.15	1.10, 1.20	**<0.0001**
**Multivariate model**						
	**SFV**			**HTLV**		
***Variable***	***Odds ratio***	***95% CI*** ^***1***^	***P value***	***Odds ratio***	***95% CI***	***P value***
**Sex**			0.07			0.124
* Female*	1.49	0.97, 2.30		1.99	0.83, 4.81	
* Male (Reference)*						
**Age at sampling (5 years increase)**	1.12	1.05, 1.18	**0.0003**	1.18	1.12, 1.25	**<0.0001**

1.CI, confidence interval.

**Familial clustering of SFV and HTLV.** We first looked at SFV infection among spouses of SFV-infected persons to gain insights on risk of SFV infection by sexual or intimate contact. There was a total of 208 spouse pairs, including five pairs with concordant SFV-positive results, 55 pairs with discordant results, and 148 pairs with concordant SFV-negative results. The five concordant SFV-positive pairs were from three tribes, including three Parakanã C, one Xikrin and one Arara do Kurambê pairs. The mean and median male and female ages of these five pairs were 36.2 and 31.0, and 33.4 and 32.0 yo, respectively. The male and female ages of the concordant SFV-positive pairs ranged from 26-60 and 19–50 yo, respectively. The 55 SFV discordant pairs were from 10 tribes (Arawete, Asuruni do Trocara, Parakanã C, Jamamadi, Mundurucu, Tiriyó, Urubu Kaapor, Waiãpi, Warao, and Xikrin). Of the 55 SFV discordant pairs, there were 27 SFV-positive males and 25 SFV-positive females and included three SFV-positive men who each had two SFV-negative wives. The male and female ages of the discordant SFV-positive pairs ranged from 23-59 and 12–59 yo, respectively. The 148 concordant SFV-negative pairs were from 13 tribes. The male and female ages for the 148 SFV-negative concordant pairs ranged from 13-84 yo and 12–79 yo, respectively. Statistical analysis of SFV infection status in concordant and discordant infection pairs with their mean ages showed no association of age and SFV infection of both males and females indicating no difference in ages in pairs with and without SFV infection.

In comparison, for HTLV there were a total of 200 spouse pairs, including 10 pairs with concordant HTLV-positive results, 41 pairs with discordant results, and 149 pairs with concordant HTLV-negative results. All 10 HTLV seroconcordant spouses were from the Xikrin tribe. The mean and median male and female ages of these 10 pairs were older than the SFV-positive concordant pairs which were 44.9 and 41.0, and 37.6 and 41 yo, respectively. Two HTLV-2-positive Xikrin men each had two HTLV-2-infected wives. Similarly, the male ages of these 10 pairs were older than those of the men in the SFV-positive concordant pairs and ranged from 35-84 yo, while the female ages were comparable to those of the women in the SFV-positive concordant pairs (22–44 yo). There were 41 HTLV discordant pairs from three tribes (Parakanã C, Urubu Kaapor, and Xikrin) and 149 pairs from 13 tribes with concordant HTLV-negative results. The 41 HTLV discordant pairs included 9 men and 27 women who were HTLV-positive and for which five women and one man each had two separate HTLV-negative spouses. The male and female ages of the 41 discordant HTLV-positive pairs ranged from 15-72 yo and 13–59 yo, respectively. The male and female ages for the 149 HTLV-negative concordant pairs from 12 tribes ranged from 13-80 yo and 12–71 yo, respectively. In contrast to SFV, for HTLV infection we found that the mean age of females when males were infected but females were not, was smallest compared to the other scenarios (*p* < 0.0001).

We then looked at SFV infection among children who had an SFV-infected mother to assess potential mother-to-child transmission. We only included children ≤ 10 yo because Amerindian culture generally supports spousal partnering above this age. There were four SFV-positive mother-child pairs in three tribes (Parakanã C (n = 1), Urubu Kaapor (n = 1), and Xikrin (n = 2)). The mean and median age of the mothers and their children were 28.3 and 27.5 and 7.3 and 7.5 yo, respectively. The age ranges of the mothers and their children were 22–36 and 4–10 yo, respectively. The children of the Parakanã C and the Urubu Kaapor were both males, while the children of the Xikrin were both females. Twenty SFV-positive mothers (mean age 30.2 yo) from 7 tribes (Arara do Kurambê, Parakanã C, Jamamadi, Mundurucu, Tiriyó, Urubu Kaapor, and Xikrin) had 42 SFV-negative children (mean age 4.8 yo, range 0.5-10 yo) with 12 positive mothers having more than one negative child (ranged from 2-5 SFV-negative children). Eight SFV-negative mothers (mean age 29.8 yo) from 5 tribes (Asuruni do Trocara, Parakanã C, Jamamadi, Mundurucu, and Xikrin) had 11 SFV-positive children (mean age 5.4 yo, range 2–9 yo) with three mothers each having two SFV-positive children. Two of the 11 children (18.2%) had the same SFV-positive father.

For comparison, we also examined HTLV-2 infection among spouses and children. Consistent with the known ability of HTLV-2 to transmit from mother to child mostly via breast-feeding, we found 11 positive mother-child pairs from two tribes (Parakanã C (n = 2) and Xikrin (n = 9)). The mean and median age of the mothers and their children were 35.7 and 41.0 and 5.7 and 5.0 yo, respectively. The age ranges of the mothers and their children were 24–44 and 2–10 yo, respectively. The children of the Parakanã C were both males from the same mother, while the children of the Xikrin were both male (n = 4) and female (n = 5), with one Xikrin mother having a son and daughter who were both HTLV-positive. Twenty-three HTLV-positive mothers (mean age 37.9 yo) from two tribes (Parakanã C and Xikrin) had 44 HTLV-negative children (mean age 4.6 yo, range 1–10 yo) with 13 mothers having more than one negative child (ranged from 2-4 HTLV-negative children). Two HTLV-negative mothers (mean age 29.5) from two tribes (Parakanã C and Xikrin) each had an HTLV-positive child who were both 10 yo.

Statistical analysis of SFV infection status in concordant and discordant infection spousal pairs with their mean ages showed no association of age and SFV infection of both males and females in these pairs. We observed the mean age of females when both spouses were not HTLV-infected was smaller then when spouses were infected (26.3, vs 38.2, 30.1, and 40.5, respectively; *p* = 0.0001). We found males were older than females regardless of their SFV or HTLV infection status (mean difference = 6.55 and 6.45 years older, respectively; *p* < 0.0001). In contrast to SFV, HTLV infections among men and women pairs were significantly associated (*p* = 0.001). When males were HTLV-infected, 46.7% of their female spouses were HTLV-infected. However, when females were infected, only 12% of their male spouses were infected. SFV and HTLV infection status were independent variables in this analysis and co-infection with both viruses was not assessed due to the small numbers of persons with both infections. The numbers of young children ≤10 yo was also too small to infer statistical associations of mother-to-child transmission of SFV and HTLV using linear regression analysis. However, we found no statistical significance between SFV and HTLV seropositivity in concordant and discordant pairs by using a two-tailed Fisher’s exact test (*p* > 0.7 for all pairs).

**Fig 3:**
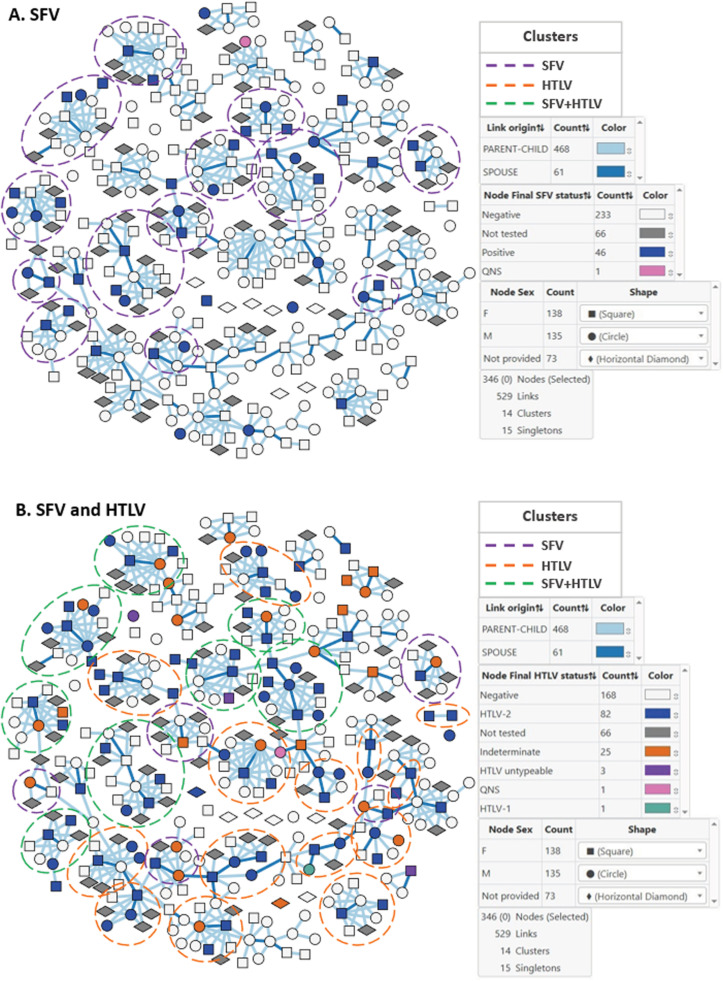
Familial clustering of simian foamy virus (SFV) and human T-cell lymphotropic virus (HTLV) infections in the Xikrin. We used MicrobeTrace (https://github.com/CDCgov/MicrobeTrace/wiki) to show clustering of infections in the Xikrin. A. SFV clustering; B. SFV and HTLV clustering. Nodes are colored by SFV or HTLV serology results and shaped by sex as shown in the key tables. Links are colored by spouse (dark blue) or parent-child (light blue) relationships. Circles around family units with dashed lines show the SFV (purple) and HTLV (orange) clusters. Dual SFV and HTLV infections are shown with dashed green circles. All possible combinations of retroviral infections are only shown for the HTLV transmission network. Singleton nodes are those that were not part of a family unit. QNS, quantity of specimen not sufficient for serologic testing.

We also evaluated familial transmission of SFV and HTLV with MicrobeTrace. Potential transmission clusters were defined by the presence of ≥ 1 seropositive result in a single family or extended family (grandparents and great-grandparents or shared spouses). We found that there were more SFV clusters (n = 25) among more tribes (n = 7) and more total members in the clusters (n = 70) compared to HTLV (n = 23, n = 2, and n = 44, respectively) (Table 3). The Xikrin had the largest number of both SFV and HTLV infections and was also the only tribe with dual SFV and HTLV infection clusters. A total of 8 clusters in the Xikrin had dual SFV and HTLV infections containing 22 and 30 members, respectively. Fig 3 shows the MicrobeTrace cluster analysis for the Xikrin as an example of these results. Altogether, this analysis further highlights SFV familial clustering. Despite finding dual infections in five tribes (ARL, GO, PV, TI, XI) we only saw clustering of dual infections in the Xikrin likely due to their higher infection rates, more study participants, and more complete familial relationship data.

**Table 3:** Greater clustering of simian foamy virus (SFV) than human T-cell lymphotropic virus (HTLV) infections in seven Amerindian tribes.

	SFV		HTLV		Dual SFV and HTLV			
Tribe	No. Clusters	No. + members in clusters	No. Clusters	No. + members in clusters	No. Clusters	No. SFV+ members in clusters	No. HTLV+ members in clusters	Dual
Arara do Kurambê	1	2	0	0	0	0	0	0
Parakanã C	4	17	1	3	0	0	0	0
Parakanã Velho	1	3	0	0	0	0	0	0
Urubú-Kaapor	1	2	0	0	0	0	0	0
Waiãpi	3	8	0	0	0	0	0	0
Warao	2	5	0	0	0	0	0	0
Xikrin	13	33	22	41	8	22	30	8
***Total***	**25**	**70**	**23**	**44**	**8**	**22**	**30**	**8**

To gain insights into possible person to person transmission of SFV, we examined whether SFV serotypes are shared among seropositive husband-wife pairs as well as mothers and their children. Of the six husband-wife pairs, five had concordant serotypes. One pair both had SFVasp-like serotypes, while four pairs had untypeable results. One pair had discordant serotypes with the wife showing untypeable results and the husband showing the SFVasp-like serotype. These data point to possible person to person transmission but require confirmation by more specific viral sequencing analysis. There were complete serotyping data on five seropositive mothers who had seropositive children less than 10 years old. Two pairs both had SFVasp-like serotypes, and three pairs had discordant serotyping results. Two pairs had untypeable and SFVasp-like results, one pair had negative and untypeable results. This serotyping pattern does not suggest SFV transmission among mothers and children, but more specific viral sequencing is required for confirmation.

## Discussion

The public health implications of SFV infections in humans remain poorly defined as previous studies surveyed healthy populations that are exposed to NHPs and lacked long-term follow-up of large numbers of SFV-infected persons [6,24]. Defining the pathogenic potential and human-to-human spread of SFV can be facilitated by the identification of populations with a high SFV prevalence. Here, we document a high SFV prevalence of 9.5% among Amerindian persons in Brazil and Venezuela which exceeded that of HTLV-2 which is endemic in this population [9,29]. We show SFV prevalence was sustained over three decades and infection originating locally from different neotropical monkeys. As observed with HTLV-2, we document familial clustering of SFV among spouses and children suggestive of human-to-human spread [46]. Our study will enable investigations of disease association and human-to-human transmission of SFV to better define the public health implications of this infection among humans.

The observed SFV seroprevalence is supported by rigorous testing with validated EIA and WB confirmation containing NWM SFV antigens. Because DNA from peripheral blood lymphocytes was not available for sequencing to determine the NHP species origin of SFV infection, we developed and used a new serotyping method and found evidence of multiple SFV variants from different NWM subfamilies co-circulating in this population. This result is consistent with Old World NHP SFV infections in humans and underscores the susceptibility of humans to infection with a very wide range of phylogenetically distinct global SFV variants [6,20]. Our finding of an SFV-infected person in 1966 in this study likely represents the oldest documented case to date. Currently, there are at least 200 persons reported globally with Old World NHP SFV infection, and few persons with NWM SFV infection in zoo and primate workers [6,25,26]. Our finding of 190 South American persons with NWM SFV infection effectively doubles the number of known SFV infections. While previously reported SFV cases were predominantly adult men such as bushmeat hunters and animal care takers, our finding that about 40% of SFV Amerindian cases are women and 12% are children provides an opportunity to study SFV outcomes in these groups [6,20,22,23,25,47].

Frequent exposures to SFV are needed to maintain a high SFV prevalence in this population. Sources of exposures typically include SFV-infected NHPs from widely practiced hunting and consumption of these animals by Amerindian persons or keeping NHPs as pets [6,20,22,23,25,47]. In South America alone, it has been estimated that between 2–5 million monkeys/year are consumed, which has increased since 1960 from highway construction in rural areas and the switch from traditional hunting methods such as bow and arrow and darts to modern weapons comprising mostly guns [27]. This practice will likely continue to fuel primary cross-species transmission of SFV. Our findings of SFV infection among spouses and children is novel and has not been previously seen in earlier studies of humans infected with Old World NHP SFV [6,20,22,23,25,47,48]. However, SFV sequencing and other epidemiologic information is needed to confirm linkage among infected spouses and children to better document human-to-human spread. If confirmed, data suggest a likely higher transmissibility of NWM SFV in humans and a role of human-to-human spread in sustaining the high SFV prevalence in this population. Confirmation of SFV spread from person to person has also important implications for broader SFV dissemination outside populations directly exposed to NHPs. Indeed, our finding of a high prevalence of SFV and HTLV infection at a Casa do Índio in Belém, which is a large urban center in northern Brazil, provides opportunities for further spread of these viruses in metropolitan areas. Amerindian persons stay for extended periods at Casas dos Índios while receiving treatment for health conditions and have been reported to have sexual contact with non-Indian persons [29]. Likewise, It has been reported that it is common practice for indigenous men to have sexual contact with non-indigenous women, including sex workers, at small towns maintained by commercial companies near the Amerindian villages [9,45].

We found infection risk with both SFV and HTLV-2 increased with age reflecting the result of continuous exposure to the viruses. Increasing age with HTLV-2 in Amerindian persons has been reported previously [41,42]. We also noted that some SFV-infected children had SFV-negative mothers suggesting different sources of infection such as direct contact with monkeys or other potential mechanisms such as wet-nursing by SFV-infected women, or tattooing/scarification rituals. Prospective studies will be important to define mechanisms of transmission among children and adults and whether they differ from those of HIV and HTLV.

The observed high SFV prevalence will help define the pathogenic potential of SFV in humans as incidence of disease in SFV-infected persons may be low, strain-dependent, and have long latency periods. SFV can be PCR-amplified and isolated from humans by co-culture from peripheral blood years after infection underscoring its ability for long persistence in the human host [6,20,47–49]. SFV has also been amplified and isolated from throat swabs and urogenital secretions in men illustrating broad tissue distribution in infected persons [48]. Prospective studies looking at disease association and secondary transmission as well as assessment of the virologic and immunologic properties of these infections are critical. Our findings of SFV and HTLV-2 co-infected persons reflects the high prevalence of both infections in this population. It will be of interest to determine if co-infection changes disease potential of either SFV or HTLV-2 as there are indications that SFV co-infection exacerbates SIV infection in macaques and STLV-1 infection of baboons increases SFV proviral loads [17,50,51].

Our study has limitations. Molecular testing by PCR and phylogenetic analysis is needed to pinpoint the NWM species origin of the SFV infections and verify linkage of the viruses among infected spouses and children. This can be accomplished with prospective studies by testing DNA from peripheral blood lymphocytes as SFV is highly cell-associated and not present in plasma [6]. Sequence analysis can also identify co-infection with different SFV strains as previously demonstrated in humans infected with SFV from macaques which, if found, could raise the possibility of viral recombination and emergence of new SFV variants [52,53]. Complete demographic data were not available for some tribes limiting the analysis. Clinical information was also not available for study participants to evaluate disease associations with retrovirus infection. Specific behavioral risks such as tattooing, scarification, etc. were also not available for persons in our study to assess their role in SFV and HTLV transmission. Specimens were not available for all tribe members to fully assess total viral prevalence in each tribe.

In summary, using validated serological assays we identified a high prevalence of NWM SFV in numerous Amerindian tribes across a broad geographic range in South America. We document persisting SFV prevalence for at least three decades and show familial clustering of the infection. Our results will facilitate epidemiological studies in this population to better understand the public health significance of human infection with SFV.

## Supporting information

S1 Table Validation of the New World monkey (NWM) simian foamy virus (SFV) recombinant Gag protein enzyme immunosorbant assay. (PDF)

S2 Table Validation of the New World monkey simian foamy virus (SFV) serotyping assay. (PDF)

S3 Table Broad seroreactivity to New World monkey simian foamy viruses (SFV) among South American Indian persons. (PDF)
