# Supplementary material for: High prevalence of simian foamy virus infection of South American Indians
Source: PLoS Pathog. 2025 Jun 9;21(6):e1013169. doi: 10.1371/journal.ppat.1013169 (PMC12180642; doi:10.1371/journal.ppat.1013169)
Supplement: S1 Table — (PDF) [file ppat.1013169.s001.pdf]

Table S1. Validation of New World monkey (NWM) simian foamy virus (SFV) recombinant Gag protein enyzme immunosorbant assay

Category or Primate Subfamily	Genera	Total	Positive (%)	Negative (%)
WB[1]-positive NWM		**57**	**56 (98.2)**	**1 (1.8)**
Atelidae	*Alouatta palliata*	1	1 (100)	0 (0)
	Alouatta seniculus	2	2 (100)	0 (0)
	Ateles belzebuth	1	1 (100)	0 (0)
	Ateles fusciceps	2	2 (100)	0 (0)
	Ateles geoffroyi	14	14 (100)	0 (0)
	Ateles paniscus	2	2 (100)	0 (0)
	Ateles species	1	1 (100)	0 (0)
Cebidae	*Callithrix jacchus*	2	2 (100)	0 (0)
	Cebus apella	24	24 (100)	0 (0)
	Saimiri bolivienis	1	1 (100)	0 (0)
	Saimiri sciureus	1	1 (100)	0 (0)
	Saimiri species	2	2 (100)	0 (0)
Pitheciidae	*Cacajao rubicundus*	2	2 (100)	0 (0)
	Pithecia pithecia	2	1 (50)	1 (50)
WB-negative NWM		**27**	**0 (0)**	**27 (100)**
Atelidae	*Ateles fusciceps*	2	0 (0)	2 (100)
	Ateles geoffroyi	1	0 (0)	1 (100)
Cebidae	*Aotus trivirgatus*	8	0 (0)	8 (100)
	Callithrix jacchus	4	0 (0)	4 (100)
	Cebus apella	1	0 (0)	1 (100)
	Saguinus oedipus	5	0 (0)	5 (100)
	Saimiri boliviensis	5	0 (0)	5 (100)
Pitheciidae	*Pithecia pithecia*	1	0 (0)	1 (100)
NWM Total		**84**	**56 (66.7)**	**28 (33.3)**
WB-positive OWMA[2] Total		**32**	**0 (0)**	**32 (100)**
Cercopithecoidea	*Chlorocebus species*	3	0 (0)	3 (100)
	Colobus angolensis	2	0 (0)	2 (100)
	Macaca silenus	2	0 (0)	2 (100)
	Macaca sylvanus	7	0 (0)	7 (100)
	Mandrillus leucophaeus	2	0 (0)	2 (100)
	Mandrillus sphinx	4	0 (0)	4 (100)
Hominoidae	*Gorilla gorilla*	4	0 (0)	4 (100)
	Pan troglodytes	2	0 (0)	2 (100)
	Pongo abelii	2	0 (0)	2 (100)
	Pongo pygmaeus	1	0 (0)	1 (100)
	P. pygmaeus x P. abelii hybrid	1	0 (0)	1 (100)
SFV-infected US primate workers[3]	*Homo sapiens*	2	0 (0)	2 (100)
WB-positive Prosimian Total		**2**	**0 (0)**	**2 (100)**
Lemuridae	*Varecia variegata*	1	0 (0)	1 (100)
	Lemur catta	1	0 (0)	1 (100)
U.S. Blood Donors Total		**102**	**0 (0)**	**102 (100)**

HIV Total		**86**	**2 (2.3)**[4]	**84 (97.8)**
HIV-1 positive	*Homo sapiens*	42	2 (4.8)	40 (95.2)
HIV-1/2 dual positive	*Homo sapiens*	44	0 (2.3)	44 (100)
HTLV Total		**47**	**0 (0)**	**47 (100)**
HTLV-1	*Homo sapiens*	8	0 (0)	8 (100)
HTLV-2	*Homo sapiens*	14	0 (0)	14 (100)
HTLV (type not provided)	*Homo sapiens*	23	0 (0)	23 (100)
HTLV-1/2 dual positive	*Homo sapiens*	2	0 (0)	2 (0)
Grand Total		**353**	**58 (16.4)**	**295 (83.6)**

1. WB, western blot. WB-positivity or-negativity using NWM or OWMA SFV antigens depending on the genera tested.
2. OWMA, Old World monkeys and apes.
3. Both primate workers were exposed to OWMA and NWM; one each was infected with baboon and chimpanzee SFV.
4. Both HIV samples reactive to NWM SFV recGag antigens were from African patients and showed high background in the SFV NWM WB assay and were seronegative in the OWMA SFV EIA and WB.
