# Supplementary material for: High prevalence of simian foamy virus infection of South American Indians
Source: PLoS Pathog. 2025 Jun 9;21(6):e1013169. doi: 10.1371/journal.ppat.1013169 (PMC12180642; doi:10.1371/journal.ppat.1013169)
Supplement: S2 Table — (PDF) [file ppat.1013169.s002.pdf]

Table S2. Validation of New World monkey simian foamy virus (SFV) serotyping assay

Subfamily	Genera	Total	SFVasp reactive (%)	SFVcja reactive (%)	SFVasp/SFVcja untypeable (%)
Atelidae	*Alouatta guariba*	1	1	0	0
	Alouatta guariba clamitans	1	1	0	0
	Alouatta palliata	1	1	0	0
	Alouatta seniculus	6	5	0	1
	Ateles belzebuth hybridus	1	1	0	0
	Ateles chamek	6	6	0	0
	Ateles fusciceps robustus	2	2	0	0
	Ateles geoffroyi	9	9	0	0
	Ateles geoffroyi vellerosus	3	3	0	0
	Ateles spp.	1	1	0	0
	Lagothrix cana	1	0	0	1
	Lagothrix lagotricha	5	1	1	3
	Total	**37**	**31 (83.8)**	**1 (2.7)**	**5 (13.5)**
Cebidae	*Aotus trivirgatus*	1	0	1	0
	Callithrix aurita	1	0	0	1
	Callithrix geoffroyi	1	0	1	0
	Callithrix jacchus	1	0	1	0
	Cebus albifrons	3	0	3	0
	Cebus apella	18	0	17	1
	Cebus robustus	1	0	1	0
	Cebus xanthosternos	1	0	1	0
	Leontopithecus chrysomelas	4	0	4	2
	Leontopithecus rosalia	2	0	2	0
	Saimiri boliviensis	3	1	2	0
	Saimiri boliviensis peruviensis	1	1	0	0
	Saimiri sciureus	4	2	1	1
	Saimiri spp.	7	3	2	2
	Saimiri ustus	4	2	2	0
	Total	**52**	**9 (17.3)**	**38 (73.1)**	**7 (13.5)**
Pitheciidae	*Cacajao melanocephalus*	6	0	5	1
	Cacajao rubicundus	3	0	3	0
	Chiropotes spp.	1	0	0	1
	Pithecia pithecia	9	2	2	5
	Total	**19**	**2 (10.5)**	**10 (52.6)**	**7 (36.8)**
	Grand Total	**108**	**49 (45.4)**	**49 (45.4)**	**19 (17.6)**

1. SFVasp, SFV from *Ateles* species (spider monkey); SFVcja, SFV from *Callithrix jacchus* (common marmoset)
