# Supplementary material for: High prevalence of simian foamy virus infection of South American Indians
Source: PLoS Pathog. 2025 Jun 9;21(6):e1013169. doi: 10.1371/journal.ppat.1013169 (PMC12180642; doi:10.1371/journal.ppat.1013169)
Supplement: S3 Table — (PDF) [file ppat.1013169.s003.pdf]

Table S3. Broad seroreactivity to New World monkey simian foamy viruses (SFV) among South American Indian persons

Tribe	Total	SFVasp reactive (%)	SFVcja reactive (%)	SFVasp/SFVcja untypeable (%)	Negative
Arara do Kurambê	3	1	0	1	1
Arara do Larangal	2	0	0	0	2
Araweté	6	2	0	2	2
Asuruni do Trocara	7	3	0	4	0
Gorotire	2	2	0	0	0
Jamamadi	5	1	0	4	0
Molokopote	1	0	0	0	1
Mundurucu	3	1	1	1	0
Parakanã C	25	1	0	23	1
Parakanã Novo	3	0	0	2	1
Parakanã Velho	12	4	3	0	5
Tiriyó	19	5	0	11	3
Urubú-Kaapor	10	3	0	7	0
Waiãpi	36	12	2	21	1
Xikrin	52	15	4	29	4
Warao	13	3	0	9	1
Casa do Índio Belém	5	1	0	4	0
Casa do Índio	5	1	0	4	0
Total	209	55	10	122	22

1. SFVasp, SFV from *Ateles* species (spider monkey); SFVcja, SFV from *Callithrix jacchus* (common marmoset)
